# Tube-Free Conjunctivodacryostomy: A Modified Technique for Refractory Proximal Lacrimal Obstruction With an Adequately Sized Lacrimal Sac

**DOI:** 10.1167/tvst.15.6.6

**Published:** 2026-06-02

**Authors:** Jiayi Zhang, Junye Zhu, Yunhai Tu, Tingting Chen, Zhenbin Qian

**Affiliations:** 1 National Clinical Research Center for Ocular Diseases, Eye Hospital, Wenzhou Medical University, Wenzhou, China; 2 The Affiliated People's Hospital of Ningbo University, Ningbo, China

**Keywords:** conjunctivodacryocystorhinostomy, tube-free, refractory proximal lacrimal obstructions, adequately sized lacrimal sac

## Abstract

**Purpose:**

This study evaluates the efficacy of a modified conjunctivodacryocystorhinostomy (CDCR) technique without the use of permanent intubation for the management of refractory proximal lacrimal obstruction (RPLO) in patients with an adequately sized lacrimal sac.

**Methods:**

A prospective interventional case series was conducted, including 16 consecutive patients (18 eyes) with RPLO and an adequately sized lacrimal sac, recruited between January 2022 and December 2024. All patients were treated with a modified tube-free CDCR technique. Anatomical and functional success rates were assessed using lacrimal irrigation and the Munk score over a follow-up period of at least 6 months.

**Results:**

The mean patient age was 52.3 ± 13.6 years, with a mean follow-up of 12.4 ± 5.6 months. The anatomical patency rate was 83.3% (15/18), and the functional success rate for epiphora was 77.8% (14/18). The postoperative Munk epiphora score of 2 (range, 1–3) was significantly lower than the preoperative score of 5 (range, 4–5) (*Z* = −3.6; *P* < 0.001). Postoperative complications included granuloma formation in four cases and occasional medial canthal discharge in five cases, with no additional complications reported.

**Conclusions:**

Modified CDCR without tube implantation demonstrated favorable clinical outcomes in patients with RPLO and an adequately sized lacrimal sac, with high anatomical and functional success rates.

**Translational Relevance:**

This approach may provide an effective surgical alternative for refractory proximal lacrimal obstruction, with the potential to decrease the occurrence of complications associated with long-term tube placement.

## Introduction

Proximal obstruction of the lacrimal drainage system, affecting the canaliculi and common canaliculus, represents a common etiology of epiphora. A range of surgical techniques, including dacryoendoscopy with intubation, laser canaliculoplasty with intubation, balloon canaliculoplasty with intubation, trephination with intubation, and dacryocystorhinostomy (DCR) with retrograde intubation, have been used for its management.^1^ Despite these techniques, a subset of patients does not achieve successful restoration of lacrimal outflow. Such cases are defined as refractory proximal lacrimal obstructions (RPLOs), which often necessitate bypass surgery. Conjunctivodacryocystorhinostomy (CDCR) with permanent tube implantation is recognized as the gold standard for the treatment for RPLO. This procedure involves the formation of a new drainage pathway between the conjunctiva and the nasal cavity, secured by permanent placement of a glass tube to ensure sustained patency.^2^

Standard CDCR demonstrates a reported surgical success rate of 50.0% to 97.5%,^3^^–^^6^ yet it is associated with a high rate and diverse range of postoperative complications, coupled with generally low patient satisfaction.^7^ Nearly all patients experience at least one postoperative complication.^8^ Commonly reported complications include tube displacement, conjunctival hyperplasia and granuloma formation, tube obstruction, infection, increased discharge, and reflux.^9^^,^^10^ Although comprehensive preoperative counseling is provided regarding long-term risks associated with a permanent Jones tube, 35% of patients still reported difficulties with tube maintenance,^8^ and 53.3% expressed dissatisfaction with the surgical outcome.^11^

Due to the numerous complications associated with permanent glass tube implantation, multiple CDCR techniques that avoid permanent tube placement have been investigated clinically. However, whether using a free oral mucosa graft around a Jones tube or a pedicled bulbar conjunctival flap around a silicone lacrimal duct stent for reconstruction of the lacrimal passage, no technique has consistently achieved favorable long-term outcomes after tube removal.^12^^,^^13^ This study presents a novel modification of CDCR without permanent intubation and reports its long-term surgical outcomes.

## Methods

### Study Design and Participants

This prospective study enrolled consecutive patients with RPLO who underwent tube-free CDCR surgery between January 2022 and December 2024 at the Eye Hospital Affiliated to Wenzhou Medical University. Ethical approval was obtained from the institutional Ethics Committee of Biomedical Research Involving Humans (Approval No. 2021-197-K-171-01), and the study complied with the Declaration of Helsinki. Written informed consent was obtained from all participants. The eligibility criteria included age 16 years or older and RPLO associated with an adequately sized lacrimal sac. RPLO was defined as either failure of canaliculoplasty with intubation or a history of two or more unsuccessful canaliculoplasties with intubation. Diagnostic imaging included orbital computed tomography scans, evaluated at a window width of 800 Hounsfield units and a window level of 200 Hounsfield units, and lacrimal sac ultrasound examination. Adequate sac size was defined by the presence of gas or fluid within the sac and vertical diameter of 12 mm or greater, anteroposterior diameter of 5 mm or greater, and transverse diameter of 5 mm or greater. Exclusion criteria included traumatic displacement of the lacrimal sac, ectropion, prior dacryocystectomy, and systemic conditions unsuitable for surgical intervention.

Postoperative follow-up was scheduled at 1, 2, 3, 6, 9, 12, and 18 months. Patients with a follow-up of less than 6 months were excluded from the analysis. Collected data included demographic characteristics, lacrimal irrigation findings, Munk score assessment, evaluation of fistula and granuloma formation, and documentation of reflux, discharge, and complications. Anatomical success was defined as passage patency on irrigation, while treatment success was defined as anatomical success combined with at least a two-grade improvement in the Munk score.^14^

### Surgical Technique

**Figure 1.**
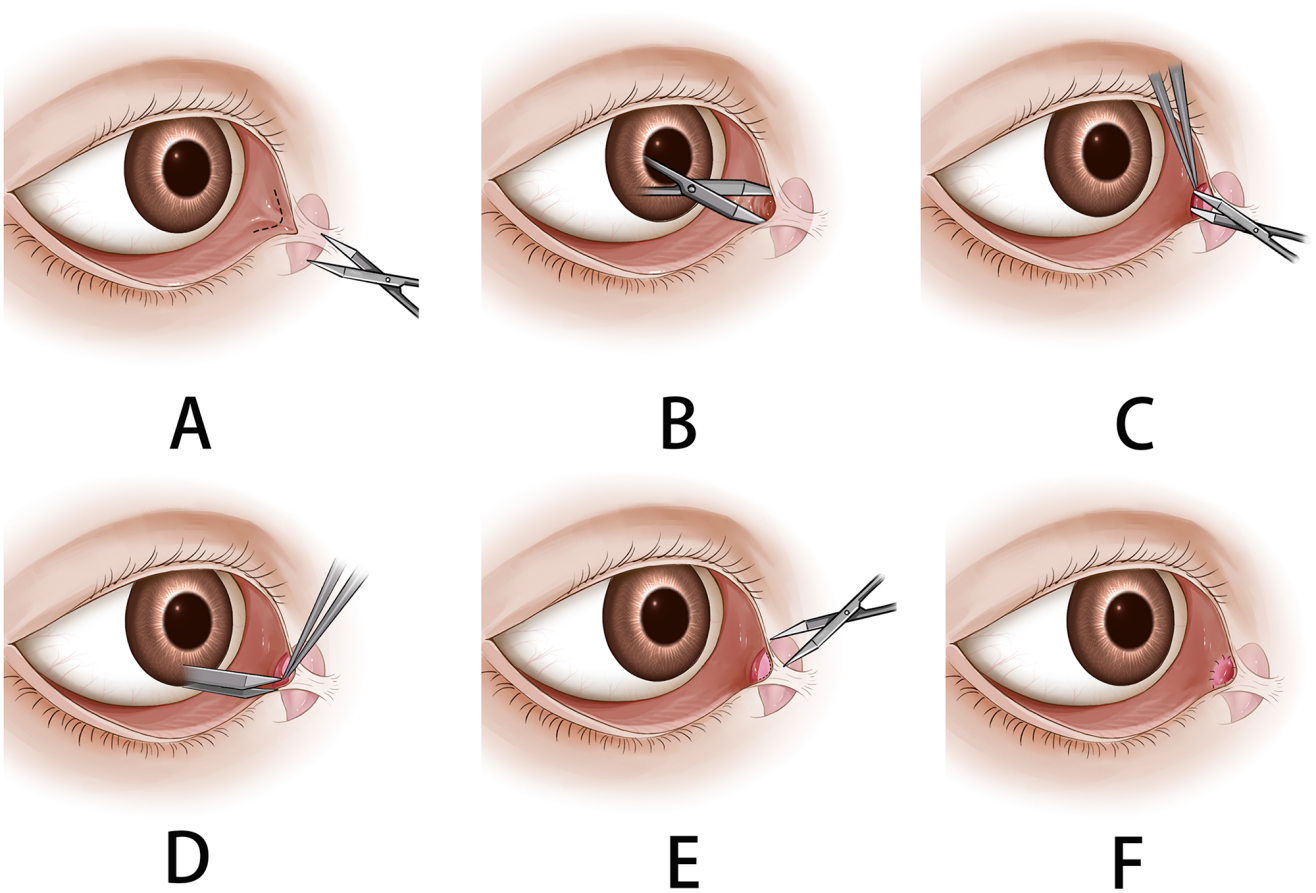
Schematic illustration of the modified tube-free CDCR surgical procedure. (**A**) Creation of the surgical access via an incision at the medial canthal conjunctival junction. (**B**) Blunt dissection of the subcutaneous tissue and Horner's muscle toward the medial orbital wall. (**C**) Dissection, excision of the subcaruncular soft tissue, and mobilization of the caruncular conjunctiva. (**D**) Excision of the mobilized Horner's muscle overlying the lacrimal sac. (**E**) Exposure of the lacrimal sac of the lacrimal sac and creation of a C-shaped mucosal flap. (**F**) Interrupted suturing of the conjunctival flap to the lacrimal sac mucosal flap.

**Figure 2.**
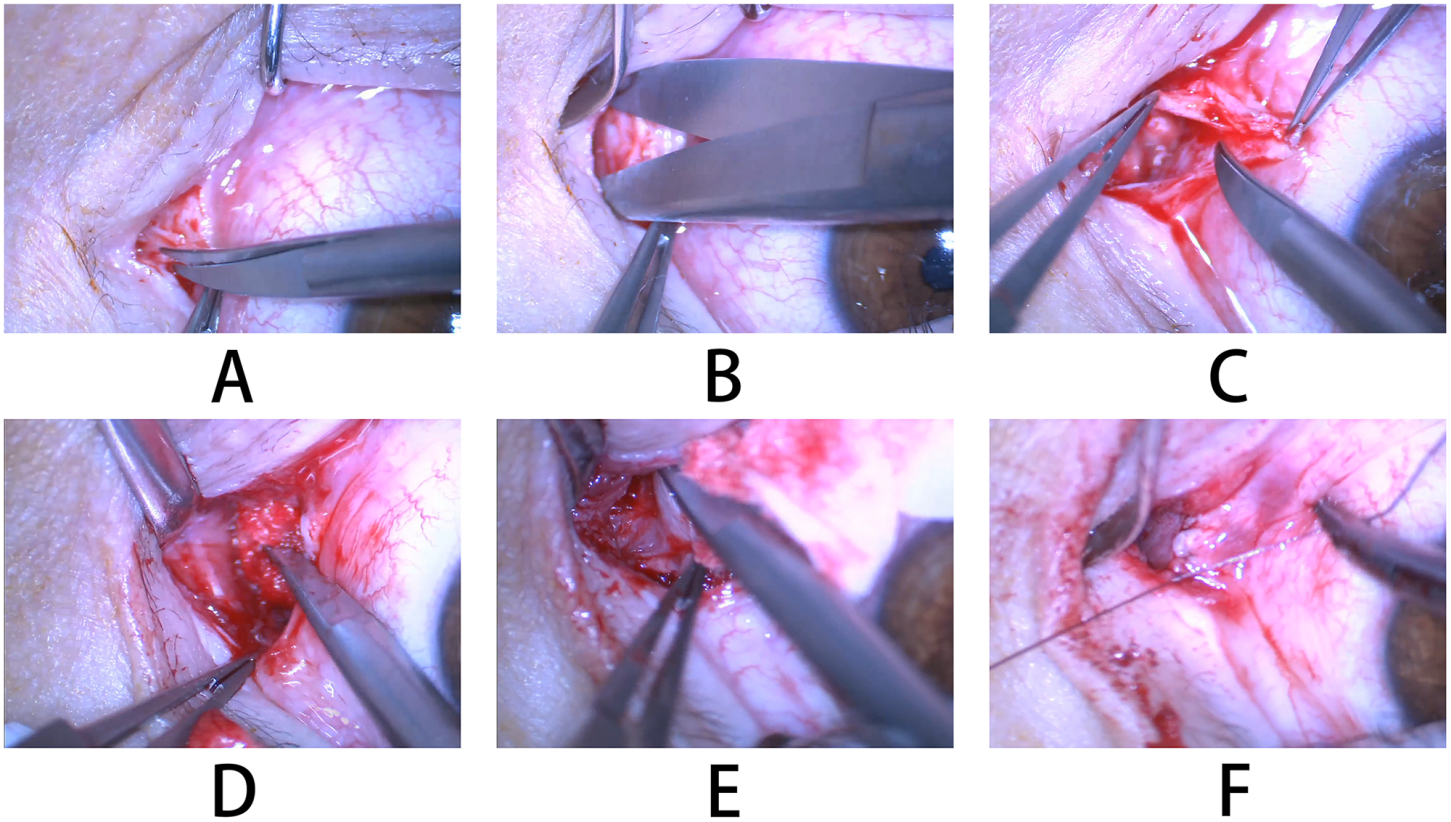
Intraoperative photographs of the modified tube-free CDCR procedure. (**A**) Creation of the surgical access at the junction of the palpebral and bulbar conjunctiva in the medial canthus. (**B**) Blunt dissection of the subcutaneous tissue and Horner's muscle toward the medial orbital wall. (**C**) Dissection, excision of the subcaruncular soft tissue, and mobilization of the caruncular conjunctiva. (**D**) Exposure of the lacrimal sac after removal of overlying Horner's muscle. (**E**) Creation of a C-shaped lacrimal sac mucosal flap. (**F**) Interrupted suturing of the conjunctival flap to the lacrimal sac mucosal flap.

All procedures were performed under general anesthesia by a senior surgeon (YT). After eyelid retraction with a speculum, the nasal skin was gently retracted using a cotton-tipped applicator. A conjunctival incision was made at the medial canthus between the palpebral and bulbar conjunctiva using Westcott scissors and extended superiorly and inferiorly for approximately 10 to 12 mm in both directions (Figs. 1A, 2A). Blunt dissection was performed along Horner's muscle toward the medial orbital wall (Figs. 1B, 2B). The caruncular conjunctiva was separated from the underlying soft tissue, and subcaruncular soft tissue was excised (Figs. 1C, 2C). Horner's muscle overlying the lacrimal sac was cleared to fully expose the lacrimal sac mucosa (Figs. 1D, 2D). The lacrimal sac mucosa was then incised in a “C”-shaped configuration with a temporal opening (Figs. 1D, 1E, 2E). The temporal flap was sutured to the caruncular conjunctiva using interrupted 8-0 polyglactin 910 sutures (Vicryl, Ethicon, Raritan, NJ), and the nasal flap was sutured to the superior and inferior palpebral conjunctiva at the medial canthus (Figs. 1F, 2F). When tension-free anastomosis was difficult, a 1- to 2-mm inferior skin incision along the medial canthal region was made to increase conjunctival mobility and reduce tension at the conjunctival–lacrimal sac flap edges. After CDCR completion, standard endoscopic DCR was performed, during which the lateral nasal mucosa over the lacrimal sac fossa was excised, and an appropriately sized osteotomy was created using an angled coarse diamond burr or forward-biting punch. The lacrimal sac was then tented through the superior canaliculus, incised vertically with a sickle knife to form a large posterior flap, and flattened against the lateral nasal wall.^15^ Throughout both procedures, the lacrimal sac was preserved without dissection or retraction.

Postoperative management included topical antibiotic-corticosteroid eye drops (Maxitrol, Alcon Laboratories, Fort Worth, TC) four times daily and intranasal corticosteroid spray (Rhinocort Aqua, AstraZeneca, Wilmington, DE) three times daily for 1 month, along with antibiotic-corticosteroid ophthalmic ointment (Maxitrol, Alcon Laboratories) once nightly for 7 days.

### Statistical Analysis

Statistical analysis was performed using IBM SPSS Statistics software (version 29.0; IBM, Armonk, NY). Descriptive statistics were used to summarize patients' clinical and demographic characteristics. The Wilcoxon signed-rank test was used to evaluate differences in epiphora severity, assessed using the Munk scale preoperatively and postoperatively. A *P* value of less than 0.05 was considered statistically significant.

## Results

A total of 17 patients underwent surgery between January 2022 and December 2024 with 16 patients (18 eyes) included in the final analysis after one loss to follow-up. The mean patients age was 52.3 ± 13.6 years, and the mean follow-up duration was 12.4 ± 5.6 months. Seven cases had at least 12 months of follow-up, and five reached at least 18 months. An intraoperative inferior medial canthal skin incision was required in three cases. Etiologies of RPLO included trauma, inflammation, idiopathic causes, chemotherapy-related factors, and congenital abnormalities are presented in Table.

**Table.** Baseline Characteristics of Patients Who Underwent Tube-Free CDCR

No	Age (Years)/Sex	Follow-Up Duration (Months)	Created Passage Irrigation	Munk Scale (Preoperative)	Munk Scale (Postoperative)	Ocular Side	Etiology	Incising the Lower Eyelid Skin	Complications
1	38/Female	18	Patent	5	2	Right	Inflammation	No	Granulation tissue formation
2	65/ Male	9	Patent	5	2	Left	Trauma	No	Discharge when blowing nose
3	70/Female	6	Patent	5	2	Right	Idiopathic	No	No
4	53/Female	6	Obstructed	5	5	Left	Idiopathic	No	No
5	68/Female	8	Patent	5	1	Left	Inflammation	No	No
6	63/Male	19	Patent	5	2	Left	Trauma	No	Discharge when blowing nose
7	57/Female	10	Patent	4	1	Right	Idiopathic	No	No
8	51/Female	6	Obstructed	5	5	Left	Idiopathic	Yes	Granulation tissue formation
9	67/Male	9	Patent	4	0	Left	Trauma	No	No
10	64/Male	11	Patent	5	1	Left	Trauma	Yes	Discharge when blowing nose
11	62/Male	11	Patent	4	3	Right	Inflammation	Yes	No
12	18/Female	12	Patent	5	2	Right	Congenital	No	Granulation tissue formation
13	27/Male	23	Patent	5	3	Left	Congenital	No	No
14	36/Female	18	Patent	4	2	Right	Chemotherapy	No	Discharge when blowing nose
15	53/Male	12	Patent	4	2	Right	Chemotherapy	No	Discharge when blowing nose
		12	Obstructed	5	4	Left		No	Granulation tissue formation
16	45/Female	21	Patent	5	1	Right	Chemotherapy	No	No
		21	Patent	5	1	Left		No	No

**Figure 3.**
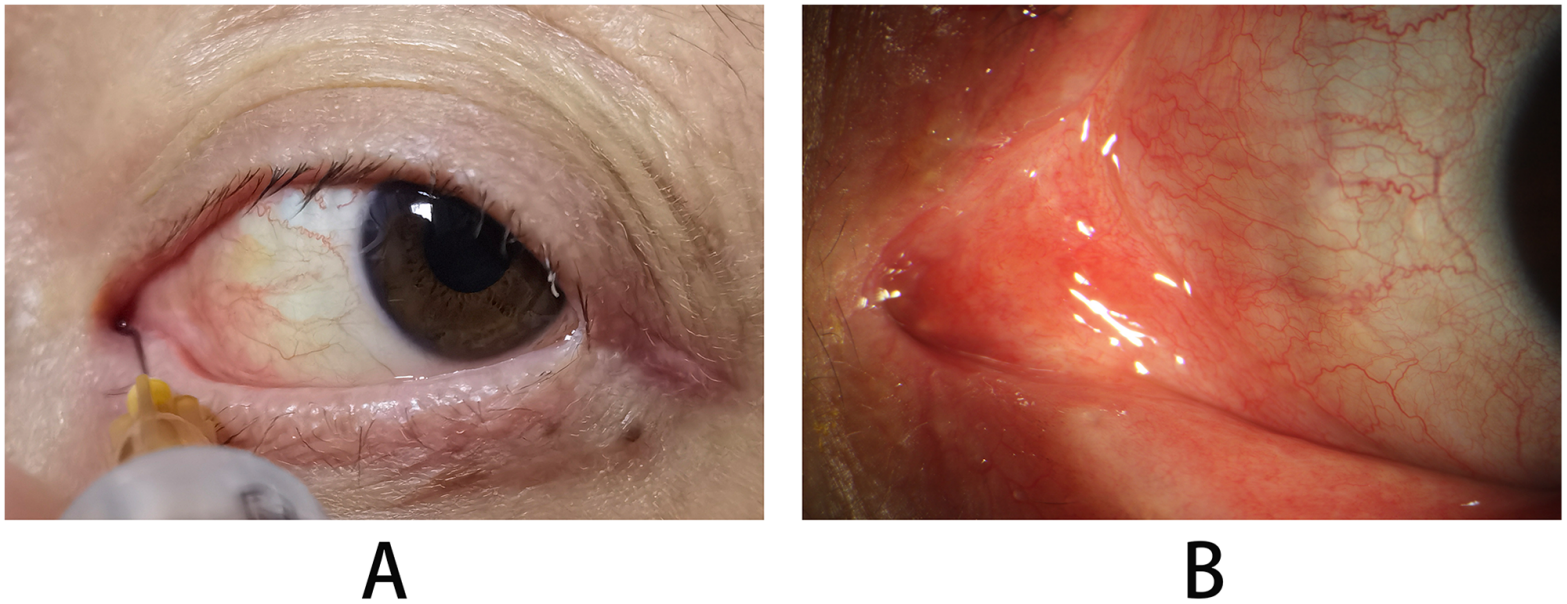
Postoperative anatomy and re-epithelialization of the conjunctivo–dacryocystorhinostomy anastomosis. (**A**) Irrigation of the newly created passage at the 9-month postoperative follow-up demonstrates patency of the anastomosis. (**B**) The anastomotic site shows complete re-epithelialization at the 1-month follow-up examination.

Final patency was achieved in 83.3% (15/18 eyes) (Fig. 3A), all of which demonstrated complete re-epithelialization (Fig. 3B). Passage narrowing was observed in all cases within 3 to 6 months postoperatively. Closure occurred in 16.7% (3/18 eyes), all at the anastomotic site due to stenosis or granulation tissue formation. Among these, one case progressed to closure at 2 months due to stenosis, and two developed granulation tissue leading to closure at 6 weeks and 3 months. Granulation tissue formation at the CDCR anastomosis was observed in 4 of the cases (22.2%), including 2 of the failed cases (66.7%) (Table).

Tearing symptoms improved in 14 of the 18 cases (77.8%), and 4 eyes showed no improvement (22.2%, 4/18). These included the three cases with anastomotic closure and one case with a patent but stenotic passage.

The Munk score significantly improved from 5 (range, 4–5) preoperatively to 2 (range, 1–3) postoperatively (*Z* = −3.6; *P* < 0.001) . No ocular motility disturbance or cosmetic dissatisfaction was reported. Among patients with a successful ostium, five cases (33.3%) reported occasional medial canthal discharge during nose blowing, with no spontaneous discharge or other complications observed (Table).

## Discussion

CDCR with permanent Pyrex glass tube implantation is widely regarded as the gold standard treatment for RPLO. Although success rates of 50.0% to 97.5% have been reported, long-term retention is associated with a broad spectrum of complications that require frequent follow-up and interventions, significantly affecting quality of life and contributing to high patient dissatisfaction rates.^4^^–^^6^^,^^8^^,^^11^ To overcome these limitations, alternative surgical strategies have been explored. Jones^2^ first attempted CDCR without permanent tube implantation in 25 cases, in which the lacrimal sac fundus was mobilized and anastomosed to the conjunctival margins of the lacrimal fossa incision. Despite this technique, most cases subsequently failed due to closure of the newly created passage.^2^ Extensive dissection required for the sac, along with exposure of the bony lacrimal sac fossa, may have intensified local inflammatory responses, and early postoperative mechanical irritation during blinking further promoted fibrosis and subsequent failure.^16^

Unlike previous techniques, this study achieved direct anastomosis between the lacrimal sac mucosa and the conjunctiva by fully using both tissues, promoting epithelialization of the newly created passage and reducing the risk of closure. The surgical design strategically exploited the anatomical proximity between the medial canthal conjunctiva and the lacrimal sac. Excision beneath the caruncle and conjunctival mobilization reduced the distance between the two structures, enabling mucosal anastomosis. When the distance remained excessive intraoperatively, a 1- to 2-mm inferonasal medial canthal skin incision was added to improve conjunctival mobility and facilitate anastomosis. This technique provided an adequate anastomotic surface while limiting tissue exposure and mechanical disruption, supporting favorable conditions. Previous studies have demonstrated that reduced tissue injury and immediate wound approximation attenuate inflammatory signaling and profibrotic cytokine release, promoting epithelial regeneration and limiting scar formation.^17^^–^^19^ Furthermore, preservation of the lacrimal sac without stripping further minimized trauma and inflammation, which also contributed to favorable postoperative re-epithelialization. Compared with free mucosal grafts, pedicled conjunctival flaps provide superior vascular support, and experimental studies by Tochigi et al.^20^ have demonstrated that pedicled nasal mucosal flaps are associated with reduced inflammation and fibrosis. Accordingly, this study proposes that the pedicled flap promotes re-epithelialization of the postoperative passage; completion of re-epithelialization inhibits scarring,^21^^,^^22^ thus increasing the surgical success rate.

Several studies have attempted to construct a conjunctival–nasal passage using autografts; however, their clinical efficacy remains uncertain. Can et al.^12^ reported the use of oral mucosa grafts wrapped around a Jones tube for lacrimal passage reconstruction. Although the tube was removed at 6 months postoperatively in 11 patients, 9 cases developed passage obstruction.^12^ Compared with pedicled flaps, free mucosal grafts lack an intrinsic vascular supply and are, therefore, more prone to ischemic compromise. Moreover, graft mobility induced by blinking may impair wound healing, by promoting chronic hypoxia, exacerbating inflammation, and delaying tissue repair.^23^^,^^24^ Histological analysis of failed grafts revealed epithelial disorganization and inflammatory cell infiltration in the surrounding connective tissue.^12^ These findings suggest that free mucosal grafts fail to facilitate rapid wound re-epithelialization, which may contribute to postoperative passage reclosure. Ouyang et al.^13^ reported favorable outcomes using a pedicled bulbar conjunctival flap wrapped around a silicone lacrimal stent, achieving success in 9 of 10 cases after stent removal at 3 months. These findings suggest improved outcomes with pedicled flaps compared with free grafts. However, micromotion between the flap and stent during blinking may still compromise healing, and the lack of long-term follow-up remains a limitation. This study demonstrated that narrowing of the newly created passage primarily occurred within 3 to 6 months postoperatively, after which no significant progression was observed. This temporal pattern differs from that observed in CDCR with permanent tube implantation, but is consistent with anastomotic narrowing after DCR.^25^

Granuloma formation was noted in two recurrent cases and was likely related to incomplete apposition between the conjunctival and the lacrimal sac mucosal flaps. This complication occurred despite the use of a lower eyelid skin incision in one case to facilitate flap approximation. Furthermore, one case demonstrated anatomical patency without functional improvement due to persistent anastomotic stenosis.

This study has several limitations, including a relatively small sample size and a short follow-up period (mean, 12.4 months), which is shorter than that typically reported in conventional CDCR studies. The restricted cohort reflects both selective inclusion criteria and reduced patient compliance with follow-up visits after postoperative symptom improvement.

## Conclusions

This study indicates that tube-free CDCR may be considered an effective and feasible surgical technique that appears to eliminate the need for permanent glass tube implantation while achieving a high rate of anatomical and functional success. In patients with an adequately sized lacrimal sac, this technique leverages the anatomical proximity between the lacrimal sac and the caruncular conjunctiva, enabling a direct anastomosis that facilitates rapid re-epithelialization of the newly created passage. Preservation of the lacrimal sac without mobilization minimizes perisac tissue disruption and helps to maintain lacrimal pump function. These findings support tube-free CDCR as a viable alternative for the management of nonreconstructable canalicular obstruction in appropriately selected patients.
